# The Roles of Anxiety and Depressive Symptoms in the Relationship Between School Bullying Victimization and Suicidal Ideation Among South Korean College Students: A Serial Multiple Mediation Model

**DOI:** 10.3390/ijerph22020256

**Published:** 2025-02-11

**Authors:** Aely Park

**Affiliations:** Department of Social Welfare, Sunchon National University, 255 Jungang-ro, Suncheon 56922, Jeonnam, Republic of Korea; apark@scnu.ac.kr

**Keywords:** school bullying victimization, suicidal ideation, anxiety, depressive symptoms, a serial multiple mediation model

## Abstract

This study examined the sequential mediating roles of anxiety and depressive symptoms in the effect of school bullying victimization on suicidal ideation. This study utilized a convenience sample of college students across South Korea, and an online survey was conducted. This quantitative research analyzed data collected from 1037 participants. The sample consisted of an equal gender distribution, with a mean age of 23.65 years (range: 18–30). Additionally, 30% of participants reported a household income of four million won or less (approximately USD $3076). More than half of the participants’ parents had attained a college education or higher. Also, multiple mediation analyses were conducted to examine whether anxiety and depressive symptoms mediated the relationship between school bullying victimization and suicidal ideation. In the results, multiple mediation analyses showed that school bullying victimization does not have a direct effect on suicidal ideation. On the other hand, school bullying victimization has an indirect effect on suicidal ideation through anxiety and depressive symptoms, controlling for covariates. Based on these research results, implications for school bullying prevention and intervention were discussed.

## 1. Introduction

According to the 2023 mortality statistics in South Korea, the annual number of adult suicides is approximately 14,000, marking an 8.3% increase from the previous year [1]. The number of individuals who have seriously contemplated suicidal ideation or planned suicide is estimated to exceed 7.6 million. While the average suicide rate among OECD countries is 10.7 per 100,000 people, South Korea records a higher rate of 27.3 per 100,000, ranking first among OECD countries [1]. Additionally, 50–70 percent of those who have contemplated suicidal ideation or planned suicide were found to have experienced at least one mental disorder, with depressive symptoms being the most prevalent condition [2]. The increase in mental health difficulties and suicide not only results in human capital loss, unemployment, and rising healthcare costs, but also leads to social losses such as widespread distress and anxiety. This highlights the urgent need for countermeasures to address this serious societal issue [3].

School bullying victimization is a global public health concern that affects millions of students each year [4]. Generally, school violence is a broad concept that includes both direct forms of bullying, such as physical harm, assault, and threats, and indirect forms of violence, such as ostracism or exclusion [5]. In South Korea, school violence is becoming increasingly prevalent, with the number of victims rising not only in middle and high schools but also in elementary schools [6]. Recently, the tragic case of a student who took his own life after experiencing school violence received media attention, highlighting the severity of this problem as an urgent social issue. Therefore, it is time to pay attention to the problem of school violence and make efforts to intervene and prevent it.

Previous studies have shown that school bullying victimization significantly affects both internalizing and externalizing symptoms. Victims of school bullying often exhibit internalizing symptoms, such as anxiety, depressive symptoms, and low self-esteem, as they internalize the distress caused by their victimization [7]. At the same time, the study shows that bullying victimization is associated with externalizing symptoms such as aggression, impulsivity, and rule-breaking behavior, as victims may externalize their distress in maladaptive ways. However, a meta-analysis suggests that the association between school bullying victimization and internalizing symptoms may be stronger than school bullying victimization and externalizing symptoms [8].

The impact of school bullying victimization extends to mental health outcomes, including anxiety and depressive symptoms, which are key psychosocial responses to victimization [9,10,11]. Anxiety characterized by worry, fear, and heightened alertness often arises from bullying victimization and may negatively affect social competence, academic performance, and interpersonal relationships [12,13]. Similarly, depressive symptoms accompanied by sadness, hopelessness, and low self-worth often emerge as victims internalize the negative experiences and messages associated with bullying [14]. In addition to direct bullying, exposure to cyberbullying has been found to increase internalizing symptoms, which are closely associated with suicidal ideation [15].

Anxiety and depressive symptoms are not only common responses to bullying but are also critical mediators in the pathway linking victimization to suicidal ideation [16,17]. Victims of school violence experience high levels of stress and anxiety, and this ongoing experience may escalate into depressive symptoms [18]. Previous studies have found that anxiety often serves as a precursor to depressive symptoms, with higher levels of anxiety tending to worsen depressive symptoms (e.g., [18,19,20]). Specifically, anxiety acts as a major triggering factor that exacerbates depression, and the two symptoms subsequently interact with each other, amplifying the psychological distress experienced by the victim [18]. These mental health consequences often create a compounded effect that heightens the risk of suicidal ideation [21,22]. Anxiety amplifies victims’ emotional distress, while depressive symptoms foster feelings of hopelessness and isolation, further increasing vulnerability [23,24]. This psychosocial distress may lead victims to perceive suicide as a way to escape their suffering [9].

There is a significant relationship between school bullying victimization and suicidal ideation, with bullied youth being significantly more likely to report suicidal ideation than non-bullied peers [25,26,27]. Moreover, previous studies indicated that demographic factors such as age, gender, parental education level, and household income are associated with suicidal ideation. For example, while suicide mortality is higher among men, suicidal ideation and attempts are more prevalent among women [28]. In a study conducted by college students comparing the suicidal ideation group with the control group, the average age of the suicidal ideation group was higher than that of the control group [29]. Also, a lower level of parental education was found to increase the risk of suicidal ideation [30] and economic stress, such as low household income, was also shown to be associated with suicidal ideation [31,32].

Although previous studies indicated the individual effects of anxiety and depressive symptoms on suicidal ideation, few have focused on their combined mediating roles in the context of school bullying victimization. Also, there is a lack of research on mechanisms that explain the relationships between school violence and suicide, which is recognized as a serious social problem in South Korea. Thus, this study addresses these gaps by exploring the combined mediating effects of anxiety and depressive symptoms, offering a novel perspective on the pathways linking school bullying victimization to suicidal ideation. The specific research hypotheses are as follows:

**Hypothesis 1 (H1):**  *School bullying victimization is positively associated with anxiety*.

**Hypothesis 2 (H2):**  *Anxiety is positively associated with depressive symptoms*.

**Hypothesis 3 (H3):**  *Depressive symptoms are positively associated with suicidal ideation*.

**Hypothesis 4 (H4):**  *Bullying victimization is expected to have a serial mediation effect, where it increases anxiety, which then leads to depressive symptoms and ultimately results in suicidal ideation*.

## 2. Methods

### 2.1. Participants and Procedure

This study conducted online survey research with a national sample of college students. The subjects were college students attending 2–3 year or 4-year universities nationwide, but those under 18 or over 30 were excluded. College students were conveniently sampled, and a self-administered survey was conducted using a structured questionnaire. The researcher worked with a survey firm to recruit participants and administer the survey. Survey invitations were distributed to potential participants via email and online platforms, with an expected response rate of 70%. After screening and consent, 1037 participants completed the survey using computers or mobile devices between 18–30 September 2019. To ensure data quality, digital fingerprinting software was used to detect fraudulent or unengaged responses, and all participants received a $5 online gift certificate. The survey firm obtained consent from eligible participants, then sent out a survey and collected the responses anonymously. Although data was collected using convenience sampling, to balance the national statistics, data was collected until the gender (female, male) and geographical location (Seoul and others) ratio of the survey participants reached nearly 50:50. Finally, a total of 1037 data were collected in September 2019. The research was approved by the Institutional Ethical Review Board (IRB) of the researcher’s university.

### 2.2. Measurement

#### 2.2.1. Suicidal Ideation

Suicidal ideation was measured using 6 items developed by Raynolds’ [33] Suicidal Ideation Questionnaire (SIQ), which is a widely used self-reporting measure. The SIQ is a questionnaire that asks college students how often they have thought about suicide attempts, suicide methods, and suicide timing (for example, ‘How often have you thought about killing yourself?’, ‘How often have you thought about how you would kill yourself?’, ‘How often have you thought about when you would kill yourself?’). Each item is composed of ‘never’ (1) to ‘almost every day’ (7), with higher scores indicating greater suicidal ideation frequency. The Cronbach’s alpha coefficient was 0.95.

#### 2.2.2. School Bullying Victimization

School bullying victimization was measured using four items: “before the age of 18, have your classmates or peers done any of the following behaviors to you?” including (1) “teased or ridiculed you by calling unpleasant nickname”, (2) “threatened or intimidated you by saying that they would not let you off easy if you did not do as they said”, (3) “scared or hit you and taken money or items from you”, (4) “frequently hit you with their hands or fists or kicked you”. The response categories were coded as ‘yes’ (1) or ‘no’ (0), and the four items were summed with higher scores indicating more severe school bullying victimization. Since the item was dichotomized, internal reliability was assessed using the Kuder–Richardson Formula 20 (KR-20), resulting in a value of 0.70.

#### 2.2.3. Anxiety

Anxiety was measured using the Korean version of the Brief Symptom Inventory-18 (BSI-18) designed by [34]. The Brief Symptom Inventory-18 (BSI-18) is a widely used measurement tool to assess psychological distress in Korean youth [35]. Anxiety, as a subscale of the Brief Symptom Inventory-18, consists of six items such as “I feel nervous and restless”, “I feel tense”, and “there are times when I am gripped by fear”. Anxiety was measured on a 5-point Likert scale (1 = not at all, 5 = very much), and a higher score indicates greater levels of anxiety. Cronbach’s alpha coefficient was 0.87.

#### 2.2.4. Depressive Symptoms

As in the case of anxiety, the Korean version of the Brief Symptom Inventory-18 (BSI-18) developed by [34] was utilized. Depressive symptoms, as a subscale of the Brief Symptom Inventory-18, consists of six items such as “no interest or curiosity in anything”, “lonely”, and “depressed mood”. Depressive symptoms were measured using a 5-point Likert scale (1 = not at all, 5 = very much so), and a higher score indicates greater levels of depressive symptoms. Cronbach’s alpha coefficient was 0.88.

#### 2.2.5. Covariates

This study accounted for demographic and socioeconomic characteristics by including the following covariates: age, gender, father’s educational level, mother’s educational level, and monthly household income. Age was measured as a continuous variable and gender was categorized as 1 for male and 0 for female. Education for father and mother was categorized as “graduated from college” (=1) or otherwise (=0), respectively. Family household income was measured in Korean Won (in ten thousands) per month.

### 2.3. Statistical Analysis

First, the researcher presented the demographic and socioeconomic characteristics of the sample, and then correlation analyses were conducted to examine the associations among the study variables. Next, using multiple mediation modeling, the researcher investigated the roles of anxiety and depression as mediators between school bullying victimization and suicidal ideation. This multiple mediation modeling was analyzed using the macro-program PROCESS 4.3, developed by [36]. This approach tested the statistical significance of the indirect effect based on a nonparametric bootstrapping procedure to overcome the inference problems through the ordinary least squares regression model and the normal distribution assumption [37]. Point estimates and confidence intervals for the indirect effects through mediators were estimated from bootstrapped samples. In addition, if the 95% bootstrap confidence interval does not include zero, it indicates that the mediation effect is statistically significant. The statistical significance of the mediation effect was analyzed based on the bias-corrected confidence interval estimated from 5000 bootstrap samples. Age, gender, parental education level, and household income were included as covariates in the multiple mediation model. All statistical analyses were performed using SPSS version 24.

## 3. Results

### 3.1. Descriptive Analyses 23

Table 1 shows socioeconomic characteristics in the sample. The sample has a balanced gender composition with 50% males and 50% females. Also, the sample has a mean age of 23.65 with a range from 18 to 30 years. Approximately 52% of the participants’ fathers graduated from college or higher, and about 46% of the participants’ mothers graduated from college or higher. About 30% of the participants have a monthly household income of four million won or less (approximately equivalent to USD $3076) in the sample.

Table 2 shows descriptive statistics and bivariate correlations of the study variables. School bullying victimization is 0.58 points on average (SD = 0.99). The mean level of anxiety is 13.32 (SD = 5.15) and the mean level of depression is 15.04 (SD = 5.54). Suicidal ideation is 9.12 points on average (SD = 5.83). The bivariate correlations show that participants who experienced school bullying victimization tend to report higher anxiety and depression, and also greater suicidal ideation frequency. For example, school bullying victimization is positively associated with anxiety (*r* = 0.24, *p* < 0.01), depression (*r* = 0.27, *p* < 0.01), and suicidal ideation (*r* = 0.17, *p* < 0.01).

### 3.2. Multiple Mediation Analyses

**Figure 1:**
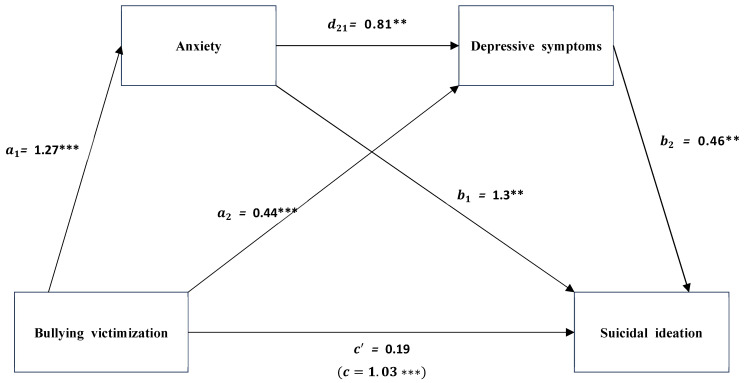
The serial mediation role of anxiety and depressive symptoms in the relationship between school bullying victimization and suicidal ideation. ** *p* < 0.01, *** *p* < 0.001.

Serial mediation analysis was conducted. The mediation analysis includes age, gender, education level of parents, and monthly household income as covariates. In Table 3, the regression results show that school bullying victimization is positively associated with anxiety ($a_{1}$ = 1.27, *p* < 0.001) and depression ($a_{2}$ = 0.44, *p* < 0.001). Anxiety and depression, respectively, are positively associated with suicidal ideation ($b_{1}$ = 0.13, *p* < 0.01; $b_{2}$ = 0.46, *p* < 0.001). Also, anxiety is positively associated with depression ($d_{21}$ = 0.81, *p* < 0.001). However, school bullying victimization is not related to suicidal ideation ($c^{\prime }$ = 0.19, *p* > 0.05). As Figure 1 shows, after including the mediators of anxiety and depression, the direct effect of school bullying victimization on suicidal ideation does not remain significant (c′).

The serial mediation analysis shows that the relationship between school bullying victimization and suicidal ideation is mediated through anxiety and depression. Table 4 shows total, direct, and indirect effects of the serial mediation model. The total effect of school bullying victimization on suicidal ideation is significant (c = 1.03, *p* < 0.001), but the direct effect of school bullying victimization on suicidal ideation does not remain significant, as explained above. Thus, the association between school bullying victimization on suicidal ideation is fully achieved through anxiety and depression. School bullying victimization is found to indirectly affect suicidal ideation through three significant pathways. The first is anxiety (*β* = 0.17, 95% CI = 0.02, 0.34), which accounted for 16.3% of the total effect, and the second is depression (*β* = 0.20, 95% CI = 0.10, 0.32), which accounted for 19.6% of the total effect. The last is anxiety and depression (*β* = 0.48, 95% CI = 0.32, 0.66), which accounted for 46.0% of the total effect. The total indirect effect was 82.0%. In other words, school violence may appear to be associated with suicidal ideation, but when anxiety and depression are accounted for, the primary pathway linking school violence to suicidal ideation is likely to occur through anxiety and depression.

## 4. Discussion

This study focused on the sequential mediating roles of anxiety and depressive symptoms in the relationship between school bullying victimization and suicidal ideation. This study found that school bullying victimization is related to suicidal ideation through anxiety and depressive symptoms, controlling for covariates. This serial multiple mediation model suggests that bullying is related to emotional stress such as anxiety, which then develops into a more serious mental health condition, depressive symptoms, and ultimately links to suicidal ideation. Therefore, it may be important to understand the underlying mechanism of the psychosocial impact of bullying through various factors in the relationship between school bullying victimization and suicidal ideation [17]. This study contributes to the development of intervention and prevention strategies to alleviate the negative impact of school violence on the mental health and well-being of youth.

Previous studies found independent associations between bullying victimization and mental health such as anxiety and depressive symptoms, or mental health and suicidal ideation. The current study developed a further understanding of the link between school bullying victimization and suicidal ideation by identifying sequential relationships using a serial multiple mediation model. This is an important step to understand the underlying mechanism between bullying victimization and suicidal ideation. To achieve this, research hypotheses were established and the results showed that school bullying victimization increased anxiety and depressive symptoms, with anxiety found to elevate depression levels, consistent with previous studies (e.g., [20,34]). In addition, higher levels of depressive symptoms were associated with an increased likelihood of suicidal ideation. These independent relationships align with findings from prior research (e.g., [9,21,22]) and, although they do not establish causality, they provide valuable insight into the pathways through which school bullying victimization may link to suicidal ideation.

These findings highlight the cumulative psychosocial impact and underscore the importance of breaking this vicious cycle through early intervention, particularly targeting anxiety and depressive symptoms [38]. It is most important to discover and intervene before the effects of school violence continue in the long term. In particular, intervention and preventive efforts targeting college students in their early adult years are necessary. Early adulthood is a period of important life cycle characteristics such as independence from parents, career choice, and employment, and is also an important developmental period for the transition to a healthy adulthood.

In terms of the practical implications, schools and communities should implement targeted interventions focused on early identification and treatment of anxiety and depression in bullying victims. First of all, school-based programs such as cognitive behavioral therapy (CBT) through school social workers can help students develop coping mechanisms to manage anxiety and depression [39]. Anti-bullying campaigns should also incorporate mental health education, emphasizing the long-term mental health impacts of bullying [40]. School social workers should assist teachers and counselors in identifying early signs of anxiety and depression in victims of school violence to ensure timely interventions. There is also a need to expand access to online resources for suicide prevention at the community level, such as self-help apps and virtual counseling [41].

Also, given that most young people in their 20s are enrolled in college in South Korea, it is crucial to identify college students with high levels of depressive symptoms and further implement active interventions. College counseling professionals should recognize childhood exposure to school violence as a significant risk factor influencing the mental health difficulties of young adults and include inquiries about such exposure during mental health evaluations. Several studies have found that victims of violence, including physical abuse and sexual violence, have relatively lower GPA scores in college, especially in the first and second years. Although school violence was not investigated, it is possible that victimization from violence is related to college grades [42,43]. Also, college students who had been exposed to violence were more likely to drop out of college than those who did not [43,44]. Thus, there is a need for universities to actively address these issues, which is essential to helping students achieve their higher educational goals.

However, this study has limitations. First, this cross-sectional study design has limitations in identifying causal relationships among variables. Cross-sectional data are collected at a single point in time, so they do not guarantee temporal order, which limits the ability to establish the temporal precedence assumption required by serial mediation models. Therefore, the results of analyses using this data should be limited to correlational relationships rather than causal relationships. Longitudinal studies may provide stronger evidence of temporal relationships among bullying victimization, anxiety, depression, and suicidal ideation. Second, the use of self-report data may introduce biases such as the possibility of under-reporting or over-reporting experiences. Also, self-report instruments may have limitations, such as social desirability bias, where respondents give answers they think are socially acceptable rather than truthful. They may also face comprehension issues if they misunderstand or misinterpret the questions due to unclear language or phrasing. Future research should consider a multi-informant approach including reports from parents, teachers, and peers. Finally, although this study emphasized the role of anxiety and depression, other potential mediators such as self-esteem, social support, and resilience should also be investigated.

## 5. Conclusions

This study examined the long-term effects of school bullying on suicidal ideation in young adulthood. The results of this study support previous studies which suggest that exposure to school bullying victimization promotes emotional vulnerability and can further lead to serious mental health problems. There were limited studies on whether experiences of school violence in childhood affect developmental outcomes in adulthood. A nationwide survey was conducted to examine how childhood bullying experiences affect adulthood. Future longitudinal studies are needed to understand the various mechanisms linking school bullying and suicidal ideation. Furthermore, schools and communities should make continued interventive and preventive efforts to address this issue.

## Figures and Tables

**Table 1:** Characteristics of participation in the survey.

	*n*	(%)
Age (Mean, *SD*)	23.65 (*2.15*)	-
Gender		
Male	518	(50.0)
Female	519	(50.0)
Father’s education (College or higher)	533	(51.6)
Mother’s education (College or higher)	476	(46.2)
Household monthly income (₩, ten thousands)		
Less than 200	53	(5.1)
200–399	275	(26.5)
400–599	298	(28.7)
600–799	191	(18.4)
800–999	113	(10.9)
More than 999	107	(10.3)

Note. *n* = 1037. There were four and six missing cases for father’s education and mother’s education, respectively, in the full sample.

**Table 2:** Descriptive statistics and bivariate correlations (N = 1037).

Variables	Test Score (Mean ± SD)	1	2	3	4
1. School bullying victimization	0.58 ± 0.99	-			
2. Anxiety	13.32 ± 5.15	0.243 **	-		
3. Depressive symptoms	15.04 ± 5.54	0.265 **	0.777 **	-	
4. Suicidal ideation	99.12 ± 5.83	0.171 **	0.457 **	0.532 **	-

** *p* < 0.01.

**Table 3:** Regression coefficients in the serial mediation analysis (N = 1030).

Path	Coefficient (SE)	95% CI		t	*p*
		Lower	Upper		
School bullying victimization → Suicidal ideation	0.186 (0.165)	−0.137	0.509	1.129	0.259
Anxiety → Suicidal ideation	0.133 (0.048)	0.039	0.227	2.762	0.006
Depressive symptoms → Suicidal ideation	0.460 (0.045)	0.372	0.548	10.290	<0.001
School bullying victimization → Depressive symptoms	0.441 (0.113)	0.219	0.662	3.907	<0.001
Anxiety → Depressive symptoms	0.812 (0.022)	0.770	0.855	37.617	<0.001
School bullying victimization → Anxiety	1.274 (0.158)	0.964	1.584	8.057	<0.001

**Table 4:** Total, direct and indirect effects of school bullying victimization (X) on suicidal ideation (Y) through anxiety (M1) and depressive symptoms (M2) (N = 1030).

Path	Estimate (SE)	95% CI ^a^		t	*p*
		Lower	Upper		
Total effects	1.034 (0.216)	0.616	1.460	4.787	<0.001
Direct effect	0.186 (0.165)	−0.137	0.509	1.129	0.259
Total indirect effect	0.848 (0.116)	0.633	1.091	-	-
Indirect effect (X → M1 → Y)	0.169 (0.080)	0.020	0.337	-	-
Indirect effect (X → M2 → Y)	0.203 (0.055)	0.102	0.317	-	-
Indirect effect (X →M1 → M2 → Y)	0.476 (0.088)	0.319	0.660	-	-

Note: Based on 5000 bootstrap samples; ^a^ 95% bias corrected confidence interval.

## Data Availability

The data is unavailable due to privacy and ethical restrictions.
